# Urinary Catheter Colonization by Multidrug-Resistant *Cedecea neteri* in Patient with Benign Prostatic Hyperplasia

**DOI:** 10.1155/2018/7520527

**Published:** 2018-07-11

**Authors:** Peter S. Ginn, Serina B. Tart, Stephen M. Sharkady, Dorothea K. Thompson

**Affiliations:** ^1^School of Pharmacy, College of Pharmacy & Health Sciences, Campbell University, Buies Creek, NC 27506, USA; ^2^Cape Fear Valley Health System, Fayetteville, NC 28304, USA; ^3^Department of Pharmaceutical Sciences, College of Pharmacy & Health Sciences, Campbell University, Buies Creek, NC 27506, USA

## Abstract

*Cedecea neteri*, a member of the *Enterobacteriaceae* family, has only been identified as a human pathogen in a few previous clinical cases, thus complicating assessment of this organism's pathogenicity and medical relevance. Documented infections attributed to *C. neteri* primarily involved bacteremia in severely immunocompromised patients. We report a rare case of urinary catheter colonization by a multidrug-resistant *C. neteri* strain in a patient of advanced age with benign prostatic hyperplasia and other chronic comorbidities. This *C. neteri* isolate was resistant or intermediate to second-generation cephalosporins, penicillins, and certain *β*-lactamase inhibitor/*β*-lactam combinations. Analysis of whole genome sequence information for a representative *C. neteri* strain indicated the presence of multiple open reading frames with sequence similarity to *β*-lactamases, including a chromosome-encoded AmpC *β*-lactamase and metallo-*β*-lactamases, consistent with the resistance phenotype of this bacterium. The presence of an AmpR homolog suggests that the *C. neteriampC* may be inducible in response to *β*-lactam exposure. Molecular insights into antibiotic resistance traits of this emerging opportunistic pathogen will be important for administering adequate antibiotic treatment to ensure favorable patient outcomes.

## 1. Introduction

The *Cedecea* genus comprises facultatively anaerobic, Gram-negative bacilli that belong to the family *Enterobacteriaceae* [1]. Formerly classified as CDC Enteric Group 15, this genus currently contains three validly described species (*Cedecea neteri*, *Cedecea lapagei*, and *Cedecea davisae*) and two unnamed species (*Cedecea* sp. 3 and *Cedecea* sp. 5) [2]. *Cedecea* species have not been reported to cause invasive infection in healthy individuals, but are considered to be opportunistic pathogens due to their clinical isolation from severely immunocompromised patients. Documented infections associated with *C. davisae* include bacteremia in patients with cancer [3], chronic renal disease, [4] and diabetes mellitus [5], as well as a scrotal abscess in an individual with chronic heart disease and alcoholic hepatitis [6]. *C. lapagei* has been reported to cause pneumonia in patients with acute promyelocytic leukemia [7], chronic obstructive pulmonary disease [8, 9], and hypoxic ischemic encephalopathy [10]. To date, *C. neteri* has been reported previously in only three documented clinical cases: a bacteremic patient with valvular heart disease [11], a systemic lupus erythematosus individual who developed an acute flare-up with bacteremia due to *C. neteri* [12], and a patient with a polymicrobial peritonitis infection following abdominal surgery [13]. In those cases involving *C. neteri*, the infection spread rapidly and caused a life-threatening situation.

We describe the first reported case of an antibiotic-resistant *C. neteri* strain isolated from the urinary catheter of an elderly patient with benign prostatic hyperplasia and chronic kidney disease. The availability of a completely sequenced *C. neteri* genome [14] presents an opportunity to explore the genetic basis of antibiotic resistance by this potentially emerging opportunistic pathogen.

## 2. Case Presentation

An 88-year-old male presented to a large community teaching hospital with a primary complaint of an irritating, generalized skin rash. The patient was afebrile. He reported recently receiving vancomycin and piperacillin-tazobactam at another area hospital for lower extremity cellulitis. Due to the extensive nature of the skin rash, he was admitted for further clinical assessment.

The patient's past medical history was significant for hypertension, benign prostatic hyperplasia (BPH), stage 3 chronic kidney disease (CKD) (baseline serum creatinine, 1.8 mg/dL), class 3 obesity (BMI 43), cholecystectomy, and left knee replacement surgery. Due to BPH progression, the patient had been using a Foley catheter for the past year which was changed monthly.

Initial laboratory results were unremarkable except for a slightly decreased red blood cell (RBC) count of 3.97 × 10^6^/*µ*L, hemoglobin 11.9 g/dL (reference range, 13.5–8.0 g/dL), and hematocrit 36.5% (40.5–54.0%). His serum creatinine was 2.02 mg/dL with an estimated glomerular filtration rate of 31 mL/min/1.73 m^2^ and elevated blood urea nitrogen (BUN) of 37 mg/dL (7–18). Urinalysis revealed a clear, yellow appearance, trace leukocyte esterase, 2+ white blood cell (WBC) count, 2+ RBC, occasional bacteria, and <1 squamous epithelial cells. An initial urine culture produced no growth after 24 hours.

The patient's skin rash, which covered more than fifty percent of his body, was treated with intravenous methylprednisolone 60 mg every 8 hours along with diphenhydramine 25 mg every 8 hours as needed. As the rash improved, the methylprednisolone was changed to oral prednisone (40 mg/day). During treatment, the patient experienced an increase in serum creatinine to 2.49 mg/dL and a BUN of 100 mg/dL. Oral prednisone was tapered to 20 mg/day, and the patient's rash improved with treatment.

During hospitalization, the patient's WBC count became elevated to 12.6, but he remained afebrile. His Foley catheter was changed, and urinalysis from the catheter was performed. Urinalysis demonstrated a cloudy, yellow appearance, 3+ leukocyte esterase, 1+ RBC, 4+ WBC clumps, and 2+ bacteria. Urine Gram stain and culture results revealed catheter colonization by Gram-negative rods with a final result of >100,000 colony-forming units/mL of *Cedecea neteri*. No other microorganism was identified from the catheter. The patient received empirical therapy of intravenous aztreonam (500 mg/8 h) until antibiotic susceptibility evaluations performed using the MicroScan WalkAway 96 Plus (Beckman Coulter) enabled de-escalation of therapy. MIC determination revealed that the *C. neteri* isolate was sensitive to piperacillin-tazobactam, ceftazidime, ceftriaxone, cefepime, aztreonam, gentamicin, tobramycin, nitrofurantoin, ciprofloxacin, and sulfamethoxazole-trimethoprim, but resistant to ampicillin, ampicillin-sulbactam, cefazolin, and cefoxitin, and intermediate to cefuroxime. The patient's WBC count returned to normal range, and therapy was de-escalated to ciprofloxacin 250 mg twice daily for 5 days prior to the patient's discharge to a rehabilitation facility.

## 3. Discussion

We describe a multidrug-resistant *C. neteri* strain isolated from the urinary catheter of an elderly patient with long-term catheterization due to progressive prostatic hyperplasia. Because *C. neteri* is capable of causing bacteremia in immunocompromised individuals [11, 12], the patient in this case was fortunate that the *C. neteri* isolate from the colonized catheter did not infiltrate the urinary tract system, causing a more serious medical condition. The source of *C. neteri* in our patient remains undetermined. However, given that the patient was using a Foley catheter during the previous year without apparent incident, it is possible that catheter colonization may have been of nosocomial origin rather than community acquired as a result of gastrointestinal colonization.

To the best of our knowledge, there are only four cases reporting the clinical isolation of *C. neteri* (Table 1), making its occurrence even less common than *C. davisae* (21 reported cases to date) and *C. lapagei* (16 reported cases to date). Two cases identified *Cedecea* species in association with ulcers (Table 1). In each case, the affected patient was either immunocompromised, had multiple comorbidities, or experienced a traumatic injury or aggressive surgical procedure, thus supporting *Cedecea* as an opportunistic pathogen.

*C. neteri* isolated from clinical specimens exhibits resistance to multiple antibiotics (Table 2). To gain insight into the potential mechanisms underlying the antimicrobial resistance phenotype, we searched the annotated genome of the representative *C. neteri* strain SSMD04 [14] for open reading frames (ORFs) with putative functions in antibiotic resistance. In silico analysis revealed a total of six chromosomal ORFs with sequence similarity to *β*-lactamases and two chromosomal ORFs with putative functions in the AmpC *β*-lactamase induction mechanism, namely ORF JT31_10465 and *ampG* (Table 3).

Based on distinctive signature motifs in the deduced amino acid sequence, we propose that ORF JT31_10470 in the SSMD04 genome is the *C. neteri bla*_*ampC*_ gene encoding the AmpC *β*-lactamase. Within the deduced 382-amino-acid sequence of JT31_10470, we identified consensus sequences, S-V-S-K (positions 85–88) and K-T-G (positions 336–338), characteristic of active-site serine *β*-lactamases [20]. Three structural elements characteristic of class C *β*-lactamases [20] and a *Cedecea davisae* AmpC [21] were also detected in JT31_10470: Y-A-N (171–173), D-A-E-A (238–241), and S-D-X-K (308–311). ORF JT31_10465 (876 bp), which contains a conserved DNA-binding helix-turn-helix (HTH) domain at the N-terminus, likely encodes AmpR, a LysR transcriptional regulator that controls expression of chromosomal *ampC* in many *Enterobacteriaceae*. The predicted *C. neteri ampC* gene (ORF JT31_10470) is located in the opposite orientation immediately upstream from the putative *ampR* gene (ORF JT31_10465), forming a divergent *ampR-ampC* operon. The presence of an AmpR homolog suggests that the *C. neteri ampC* gene may be inducible in response to *β*-lactam exposure. Consistent with the production of an AmpC *β*-lactamase, *C. neteri* clinical isolates display resistance to ampicillin, amoxicillin, first-generation cephalosporins, and cefoxitin, and are not inhibited by the *β*-lactamase inhibitors clavulanic acid and sulbactam, but are sensitive to ceptazidime, ceftriaxone, cefepime, aztreonam, and piperacillin, all of which constitute weak substrates for hydrolysis by AmpC *β*-lactamases [17].

The SSMD04 genome also contains four genes encoding putative class B metallo-*β*-lactamases (MBLs), which require zinc as a cofactor for *β*-lactam hydrolysis. For three of these ORFs (JT31_00700, 16535, and 22070), we identified the highly conserved group B-specific element H-X-H-X-D (Table 3), which is required for activity of class B *β*-lactamases [20]. MBLs catalyze the hydrolysis of a wide range of *β*-lactams, including carbapenems, whereas monobactams, such as aztreonam, are typically poor substrates for these enzymes [19]. However, *C. neteri* isolates reported in this case and in the literature were not assessed for carbapenem susceptibility. In addition, MBLs are not inhibited by clavulanic acid or tazobactam [19]. The overlapping hydrolysis profiles of metallo-*β*-lactamases and AmpC *β*-lactamases suggest that the antibiotic resistance phenotype of *C. neteri* could be due to the expression of either class of *β*-lactamases or a combination of both. It is noteworthy that the genome of another sequenced *C. neteri* strain, M006 [22], also contains multiple metallo-*β*-lactamase genes and a putative *ampC*, which exhibits 93% amino acid sequence identity to the SSMD04 *ampC* (data not shown). In-depth functional studies, which are beyond the scope of this report, are needed to verify the specific resistance mechanisms in *C. neteri*.

Clinical *C. neteri* isolates have been reported to show resistance to colistin [11], a polypeptide of the polycationic polymyxin drug class. Genes conferring colistin resistance are associated with lipopolysaccharide (LPS) modification via cationic substitution. In Gram-negative bacteria, the PhoQ/PhoP two-component system activates expression of the *pmrCAB* operon, which encodes proteins responsible for cationic modifications of LPS [18]. Homology searches revealed that the *C. neteri* SSMD04 genome contains *mgrB*, *phoP*, *phoQ*, and the *pmrCAB* operon associated with acquired colistin resistance. No plasmid DNA was found in SSMD04, ruling out the possibility of colistin resistance conferred by the plasmid-derived *mcr1* (mobilized colistin resistance) gene. However, none of the LPS-modifying genes in strain SSMD04 harbored mutations known to confer polymyxin resistance [18].

In conclusion, we report a rare case of catheter colonization by an antibiotic-resistant *C. neteri* strain. Genomic analysis of a representative sequenced strain identified a chromosomal AmpC *β*-lactamase gene that may be under induction control by an AmpR homolog, as well as the presence of multiple metallo-*β*-lactamase genes. Further research investigating the antibiotic resistance mechanisms of *C. neteri* is warranted given its increasing incidence of isolation and clinical association with severely immunocompromised patients.

## Figures and Tables

**Table 1:** Reported clinical cases involving *Cedecea neteri* and *Cedecea* sp.

Patient (age/sex)	*Cedecea* sp.	Infection	Medical history	Sensitivity	Resistance	Reference
88/M	*C. neteri*	Colonized catheter	Cellulitis, hypertension, benign prostatic hyperplasia, chronic kidney disease	Piperacillin/tazobactam, cefmandole, ceftazidime, ceftriaxone, cefepime, aztreonam, nitrofurantoin, ciprofloxacin, TMP/SMX	Ampicillin/sulbactam, cefazolin, cefoxitin	Current case

62/M	*C. neteri*	Bacteremia	Valvular heart disease	Cefamandole, chloramphenicol, tetracycline, gentamicin, tobramycin, amikacin	Cefalothin, ampicillin, colistin	[11]

27/F	*C. neteri*	Bacteremia	SLE	Vancomycin	Amoxicillin, amoxicillin/clavulanic acid, aminoglycosides, cephalosporins	[12]

NA	*C. neteri* and *Escherichia vulneris*	Peritonitis	Aggressive abdominal surgery	NA	NA	[13]

79/M	*Cedecea* sp.	Cutaneous ulcer	DM	Minocycline	NA	[15]

20/M	*Cedecea* sp.	Orbital cellulitis, corneal ulcer	Motor vehicle accident	NA	NA	[16]

M, male; F, female; NA, not available; TMP/SMX, trimethoprim/sulfamethoxazole; DM, diabetes mellitus; SLE, systemic lupus erythematosus.

**Table 2:** Antibiotic resistance patterns of *Cedecea neteri* isolated from a patient's catheter (current case) and reported in previous studies.

Antibiotic	Susceptibility (MIC, *μ*g/mL)^*∗*^	Reference number	Resistance mechanism encoded in *C. neteri* SSMD04 genome^†^
Aminobenzyl-penicillin			
Amoxicillin	*R* (>16)	[12]	AmpC^‡^, MBL^§^
Ampicillin	*R* (>16)	[11], current case	AmpC, MBL

*β*-Lactam/*β*-lactamase inhibitors			
Amoxicillin-clavulanate	*R* (8/4)	[12]	AmpC, MBL
Ampicillin-sulbactam	*R* (>16/8)	Current case	AmpC, MBL

Cephalosporins (1st generation)			
Cefazolin	*R* (>16)	Current case	AmpC, MBL
Cephalothin	*R* ^¶^	[11]	AmpC, MBL

Cephalosporins (2nd generation)			
Cefoxitin	*R* (>16)	Current case	AmpC, MBL
Cefuroxime	*I* (16)	Current case	AmpC, MBL

Polymyxins			
Colistin	*R* ^¶^	[11]	LPS modification system^#^

^*∗*^Intermediate (*I*): likely to respond to high dosage therapy. Resistant (*R*): unlikely to respond to high dosage therapy. ^†^Reference [14]. ^‡^Analysis of open reading frame (ORF) JT31_10470 (1149 bp, 382 amino acids) in the *C. neteri* SSMD04 genome indicated sequence homology to AmpC *β*-lactamases. AmpC enzymes belong to the class C cephalosporinases (reviewed in [17]). Scrutiny of the deduced amino acid sequence of JT31_10470 revealed the presence of the following conserved sequence elements characteristic of class C *β*-lactamases: S-X-S-K (positions 85 to 88), Y-A-N (positions 171 to 173), and K-T-G (positions 336 to 338). ^§^Metallo-*β*-lactamase. ^¶^Resistance phenotype was determined using the Kirby-Bauer disk method as reported in the cited reference. ^#^Components of the LPS modification system (*mgrB*, *phoP*, *phoQ*, and the *pmr* operon) are present in the annotated genome of *C. neteri* SSMD04, but these loci do not contain mutations known to confer colistin resistance [18].

**Table 3:** *β*-Lactamases and other *β*-lactam resistance proteins encoded in the *Cedecea neteri* SSMD04 genome.

Locus tags^*∗*^	Annotated gene products	Molecular class^†^	Coding genes	Sequence signatures (amino acid positions)
JT31_00700	Metallo-*β*-lactamase	B	*bla*_*MBL*_	T-H-x-H-x-D-H-x-G-G (128–137)^‡^

JT31_03975	Metallo-*β*-lactamase	B	—	S-x-x-K (72–75)^§^

JT31_07450	Muropeptide transporter, *β*-lactamase induction signal transducer	NA	*ampG*	Major Facilitator Superfamily (MFS) domains (17–366, 319–481)

JT31_10465	LysR family transcriptional regulator	NA	*ampR* ^¶^	HTH domain (8–67); LysR substrate-binding domain (91–289)

JT31_10470	*β*-Lactamase	C	*ampC* ^¶^	S-x-S-K (85–88)
				Y-A-N (171–173)
				S-D-N-K (308–311)
				K-T-G (336–338)

JT31_16535	Metallo-*β*-lactamase	B	*bla*_*MBL*_	H-x-H-x-D-H (156–161)

JT31_22070	Metallo-*β*-lactamase	B	*bla*_*MBL*_	H-x-H-x-D-H (131–136)

JT31_22350	*β*-Lactamase	A/D	—	S-x-x-K (144–147)

^*∗*^KEGG Genome database, *Cedecea neteri* strain SSMD04 (http://www.genome.jp/kegg-bin/show_organism?org=cnt). ^†^The molecular classification of *β*-lactamases is based on the primary amino acid sequence of these enzymes (also known as the Ambler classification scheme). For a review, see [19]. NA, not applicable. ^‡^Sequence signature contains the core H-X-H-X-D motif characteristic of class B *β*-lactamases, but also contains additional residues reported in [20] that constitute an expanded motif specific to metallo-*β*-lactamases. ^§^Only one conserved sequence element was identified in the deduced amino acid sequence of ORF JT31_03975. Motif S-X-X-K is characteristic of class A and D *β*-lactamases. The absence of the conserved zinc ion interaction domain (H-X-H-X-D) suggests that this ORF may encode a novel metallo-*β*-lactamase or a variant class A or D *β*-lactamase. ^¶^Proposed gene name for this *C. neteri* SSMD04 ORF based on sequence analysis (this study).

## References

[1] Grimont P. A. D., Grimont F., Farmer J. J., Asbury M. A. (1981). *Cedecea davisae* gen. nov., sp. nov. and *Cedecea lapagei* sp. nov., new *Enterobacteriaceae* from clinical specimens. *International Journal of Systematic Bacteriology*.

[2] Brenner D. J., Krieg N. R., Staley J. T., Garrity G. M. (2005). Cedecea. *Bergey’s Manual of Systematic Bacteriology*.

[3] Abate G., Qureshi S., Mazumder S. A. (2011). *Cedecea davisae* bacteremia in a neutropenic patient with acute myeloid leukemia. *Journal of Infection*.

[4] Peretz A., Simsolo C., Farber E., Roth A., Brodsky D., Nakhoul F. (2013). A rare bacteremia caused by *Cedecea davisae* in patient with chronic renal disease. *American Journal of Case Reports*.

[5] Dalamaga M., Pantelaki M., Karmaniolas K., Matekovits A., Daskalopoulou K. (2008). Leg ulcer and bacteremia due to *Cedecea davisae*. *European Journal of Dermatology*.

[6] Bae B. H., Sureka S. B. (1983). *Cedecea davisae* isolated from scrotal abscess. *Journal of Urology*.

[7] Lopez L. A. S., Ibarra B. S., de la Garza J. A. C., Rada Fde J., Nuñez A. I. S., López M. G. R. (2013). First reported case of pneumonia caused by *Cedecea lapagei* in America. *Brazilian Journal of Infectious Diseases*.

[8] Hong S. K., Lee J. S., Kim E. C. (2015). First Korean case of *Cedecea lapagei* pneumonia in a patient with chronic obstructive pulmonary disease. *Annals of Laboratory Medicine*.

[9] Yetkin G., Ay S., Kayabaş U., Gedik E., Güçlüer N., Çalişkan A. (2008). A pneumonia case caused by *Cedecea lapagei*. *Mikrobiyoloji Bulteni*.

[10] Kury C. M. H., Yabrudi A. A., de Souza T. H. (2017). First reported case of ventilator-associated pneumonia and sepsis caused by *Cedecea lapagei* in a Brazilian neonatal intensive care unit. *Journal of the Pediatric Infectious Diseases Society*.

[11] Farmer J. J., Sheth N. K., Hudzinski J. A., Rose H. D., Asbury M. F. (1982). Bacteremia due to *Cedecea neteri* sp. Nov. *Journal of Clinical Microbiology*.

[12] Aguilera A., Pascual J., Loza J. (1995). Bacteraemia with *Cedecea neteri* in a patient with systemic lupus erythematosus. *Postgraduate Medical Journal*.

[13] Anon M. T., Ruiz-Velasco L. M., Borrajo E., Giner C., Sendino M., Canton R. (1993). *Escherichia vulneris* infection. Report of 2 cases. *Enfermedades Infecciosas y Microbiología Clínica*.

[14] Chan K. G., Tan K. H., Yin W. F., Tan J. Y. (2014). Complete genome sequence of *Cedecea neteri* strain SSMD04, a bacterium isolated from pickled mackerel sashimi. *Genome Announcements*.

[15] Hansen M. W., Glupczynski G. Y. (1984). Isolation of an unusual *Cedecea* species from a cutaneous ulcer. *European Journal of Clinical Microbiology*.

[16] Clark J. D., Fernandez de Castro J. P., Compton C., Lee H., Nunery W. (2016). Orbital cellulitis and corneal ulcer due to *Cedecea*: first reported case and review of the literature. *Orbit*.

[17] Jacoby G. A. (2009). AmpC beta-lactamases. *Clinical Microbiology Reviews*.

[18] Poirel L., Jayol A., Nordmann P. (2017). Polymyxins: antibacterial activity, susceptibility testing, and resistance mechanisms encoded by plasmids or chromosomes. *Clinical Microbiology Reviews*.

[19] Munita J. M., Arias C. A. (2016). Mechanisms of antibiotic resistance. *Virulence Mechanisms of Bacterial Pathogens*.

[20] Singh R., Saxena A., Singh H. (2009). Identification of group specific motifs in Beta-lactamase family of proteins. *Journal of Biomedical Science*.

[21] Ammenouche N., Dupont H., Mammeri H. (2014). Characterization of a novel AmpC β-lactamase produced by a carbapenem-resistant *Cedecea davisae* clinical isolate. *Antimicrobial Agents and Chemotherapy*.

[22] Chan K. G., Tan W. S. (2017). Insights into *Cedecea neteri* strain M006 through complete genome sequence, a rare bacterium from aquatic environment. *Standards in Genomic Sciences*.

